# The complete mitochondrial genome of the marine feather duster, *Bispira melanostigma* (Annelida: Sabellidae)

**DOI:** 10.1080/23802359.2021.2008840

**Published:** 2021-12-15

**Authors:** Caroline J. Hornfeck, Taber C. Faurie, Logan L. Hodges, Alexis M. Janosik, Viktoria E. Bogantes

**Affiliations:** Department of Biology, University of West Florida, Pensacola, FL, USA

**Keywords:** *Bispira melanostigma*, polychaete, mitochondrial genome, mtDNA

## Abstract

The marine feather duster, *Bispira melanostigma* (Schmarda, 1861), is a tube-dwelling annelid that contributes to ecological and biogeochemical processes in benthic communities. Due to the lack of scientific data, *B. melanostigma* is often difficult to distinguish from other species of marine worms through morphological characteristics alone. In this study, we report the complete mitochondrial genome of *Bispira melanostigma.* The complete mitogenome contained 20,624 bp length with a total of 13 protein-encoding genes, 21 tRNA, and 2 rRNA genes. Phylogenetic analysis of the complete mitochondrial DNA of *B.melanostigma* can aid in the understanding of evolutionary relationships within Sabellidae.

Feather duster worms (Sabellidae) are marine tube-dwelling annelids that are common in benthic environments in marine ecosystems. As ecosystem engineers, feather duster worms alter sediment properties in close proximity by constructing tubes and burrows affecting sediment parameters such as grain size composition and porosity (Callaway 2006), creating suitable habitats for algae, and small marine organisms. Tube worms also influence biodiversity and the abundance of meiofauna by creating biogenic structures, increasing habitat complexity, and thus protection from predators (Passarelli et al. 2012). Additionally, the tube can function as a surface for larval settlement (Callaway 2006). Sabellids are easily distinguished from other marine worms, due to the presence of a feather-like appendage that projects outside of the tube (Bok et al. 2016), although identification to the species level can be challenging because of the morphologically uniform characteristics within the group (Capa et al. 2021).The focus of this study, *B. melanostigma* Schmarda, 1861) is suspension-feeder that is often misidentified due to variability within species and the lack of scientific papers on morphology. *Bispira melanostigma* is distributed globally but commonly found in the Gulf of Mexico, Caribbean, and waters surrounding Bermuda (Knight-Jones and Perkins 1998). Currently, 35 species comprise the genus *Bispira* making genomic analysis and sequencing imperative for the differentiation of species and understanding of evolutionary relationships. In this study, tissue samples were extracted and sequenced for the complete mitochondrial genome of the marine worm *Bispira melanostigma,* using Illumina Hiseq next-generation sequencing.

*Bispira melanostigma* was collected from the intertidal zone of Apalachee Bay, Wakulla County, Florida, United States (30°04′03.7″N, 84°08′22.5″W) and preserved in 200 proof ethanol. Genomic DNA of *B. melanostigma* was extracted using the DNeasy Blood and Tissue Kit (Qiagen, Valencia, CA) following the manufacturer’s standardized protocol. The specimen was deposited in the invertebrate collection at the Florida Museum of Natural History (www.floridamuseum.ufl.edu, John D. Slapcinsky, slapcin@flmnh.ufl.edu) under voucher number Annelida 009278. Library preparation of total genomic DNA was prepared using the Kapa HyperPlus kit. Samples were sequenced with 250 bp paired-end reads on Illumina Hiseq2500 platform at the Hubbard Center for Genomics, Sequencing Core Facility (Durham, NH). DNA raw reads were assembled using the *de novo* assembly method in Geneious Prime V. 2021.0.3 (https://www.geneious.com). Protein coding (PCGs) genes from complete mitochondrial genomes of Sabellidae and closely related taxa were retrieved from GenBank for phylogenetic reconstruction. PCGs used for the tree were from *Hydroides norvegica* MT919975.1*, Spirobranchus giganteus* NC032055.1*, Sabella spallanzanii* MW002660.1*, Manayunkia occidentalis* NC050262.1*, Osedax rubiplumus* MT108937.1*, Oasisia alvinae* NC026859.1*, Paraescarpia echinospica* NC037085.1*. Marenzelleria neglecta* MK120303.1 was used as the outgroup. A maximum-likelihood phylogenetic tree was constructed using MEGA-X (Kumar et al. 2018) with 1000 bootstrap replicates (Figure 1). Annotation of the assembled genome was conducted using MITOS2 (Bernt et al. 2013).

**Figure 1.**
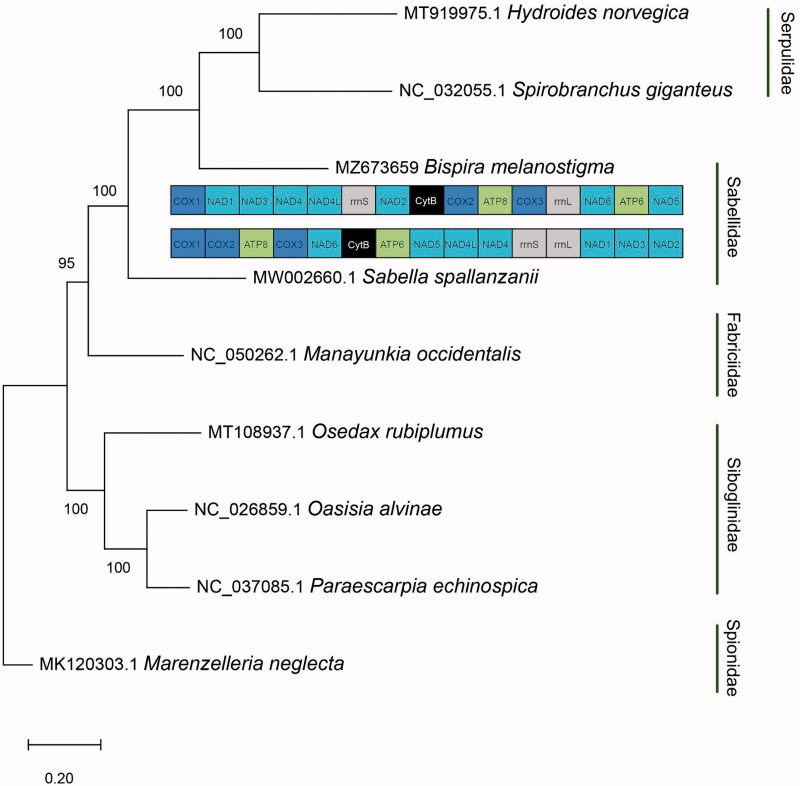
Maximum likelihood phylogenetic tree based on concatenated sequences of protein coding genes in the families Sabellidae, Serpulidae, Siboglinidae, and Fabriciidae with *Marenzelleria neglecta* as the outgroup. Bootstrap values are indicated for each node. Mitochondrial gene order is shown for *Bispira melanostigma* and *Sabella spallanzanii*.

The mitochondrial genome of *B. melanostigma* contained 20,624 basepairs in length (GenBank accession number: MZ673659), with an an overall base composition of 21.70% (A), 12.93% (C), 28.43% (G), 36.94% (T). This mitogenome consisted of a higher AT-rich content of 58.64% and a lower GC content of 41.36% and was relatively large compared to other annelid mt genomes. There was a total of 13 protein-encoding genes, 21 tRNA, and 2 rRNA genes. Notably, trnR was not recovered as part of *B. melanostigma* mitogenome. All coding regions for *B. melanostigma* were present on the positive strand, unlike, *Sabella spallanzanii* which contained all encoding genes on the negative strand except gene *nad3.*

Phylogenetic results recovered *B. melanostigma* as sister taxon to serpulids, supporting previous hypotheses suggesting that Sabellidae and not Fabriciidae is more closely related to Serpulidae (Tilic et al. 2020; Daffe et al. 2021). However, Sabellidae was not recovered as a monophyletic group (Figure 1). Interestingly, *B.melanostigma* mitochondrial gene arrangement differed highly from other annelids previously described, including the only other sabellid for which mt genome is known (Daffe et al. 2021). Only one block (*cox2-atp8-cox3*) follows the putative ground pattern for Errantia and Sedentaria within annelids (Weigert et al. 2016). These results suggest that mitochondrial gene order is not conserved among sabellids, while highlighting the need for additional mitochondrial genome sequencing of sabellids to further understand the evolution of its mitochondrial genome.

## Data Availability

The data that support the findings are openly available in NCBI at (https://www.ncbi.nlm.nih.gov/), reference number (MZ673659). The associated BioProject, SRA, and Bio-Sample numbers are PRJNA731158, SRP320569, and SAMN20122379.

## References

[CIT0001] Bernt M, Donath A, Jühling F, Externbrink F, Florentz C, Fritzsch G, Pütz J, Middendorf M, Stadler PF. 2013. MITOS: improved de novo Metazoan Mitochondrial Genome Annotation Mol. Mol Phylogenet Evol. 69(2):313–319. 22982435 10.1016/j.ympev.2012.08.023

[CIT0002] Bok MJ, Capa M, Nilsson D. 2016. Here, there and everywhere: the radiolar eyes of fan worms (Annelida, Sabellidae). Integr Comp Biol. 56(5):784–795. 27453305 10.1093/icb/icw089

[CIT0003] Callaway R. 2006. Tube worms promote community change. Mar Ecol Prog Ser. 308:49–60.

[CIT0004] Capa M, Kupriyanova E, Nogueira JM, de M, Bick A, Tovar-Hernández MA. 2021. Fanworms: yesterday, today and tomorrow. Diversity. 13(3):130–204.

[CIT0005] Daffe G, Sun Y, Ahyong ST, Kupriyanova EK. 2021. Mitochondrial genome of *Sabella spallanzanii* (Gmelin, 1791) (Sabellida: Sabellidae). Mitochondrial DNA B Resour. 6(2):499–501. 33628903 10.1080/23802359.2021.1872431 PMC7889199

[CIT0006] Knight-Jones P, Perkins TH. 1998. A revision of *Sabella*, *Bispira* and *Stylloma* (Polychaeta: Sabellidae). Zool J Linn Soc. 123:385–467.

[CIT0007] Kumar S, Stecher G, Li M, Knyaz C, Tamura K. 2018. MEGA X: molecular evolutionary genetics analysis across computing platforms. Mol Biol Evol. 35(6):1547–1549. 29722887 10.1093/molbev/msy096 PMC5967553

[CIT0008] Passarelli C, Olivier F, Paterson DM, Hubas C. 2012. Impacts of biogenic structures on benthic assemblages: microbes, meiofauna, macrofauna and related ecosystem functions. Mar Ecol Prog Ser. 465:85–97.

[CIT1317933] Schmarda LK. 1861 Neue Wirbellose Thiere: Beobachtet und gesammelt auf einer Reise um die Erde 1853 bis 1857. Turbellarien, Rotatorien und Anneliden. Part II. 164 pp.

[CIT0009] Tilic E, Atkinson SD, Rouse GW. 2020. Mitochondrial genome of the freshwater annelid *Manayunkia occidentalis* (Sabellida: Fabriciidae). Mitochondrial DNA B Resour. 5(3):3295–3315. 33458144 10.1080/23802359.2020.1815604 PMC7782465

[CIT0010] Weigert A, Golombek A, Gerth M, Schwarz F, Struck TH, Bleidorn C. 2016. Evolution of mitochondrial gene order in Annelida. Mol Phylogenet Evol. 94(Pt A):196–206. 26299879 10.1016/j.ympev.2015.08.008

